# Antenna Current Calculation Based on Equivalent Transmission Line Model

**DOI:** 10.3390/mi13050714

**Published:** 2022-04-30

**Authors:** Shusheng Wei, Wusong Wen

**Affiliations:** 1 Department of Electrical Engineering, Tsinghua University, Beijing 100084, China; weiss13@mails.tsinghua.edu.cn; 2 Key Laboratory of Military Special Power Supply, Army Engineering University of PLA, Chongqing 400035, China

**Keywords:** antenna current, transmission line model, frequency-selective surface analytical approximation

## Abstract

This paper provides a new way for spatial current/field profiles for frequency-selective surface analytical approximation. It confirms that the per unit length radiation resistance of an equivalent transmission line model for line antenna has little influence on the normalized current distribution. The two-wire equivalent transmission line model (typically used for transmitting line antenna) is applied to the receiving line antenna. In this case, the corresponding incident field is decomposed into odd and even mode for asymmetric distribution. A one-wire equivalent transmission line model is then introduced for any antenna composed of relative narrow strips. The incident field does not need to be decomposed. According to the simulation, the transmission line loss has little influence on the current distribution.

## 1. Introduction

Numerical methods, such as method of moment (MoM), are usually applied to calculate the transmitting current of dipole antenna. However, the current distribution calculated is in numerical form, which cannot be directly used for frequency-selective surface (FSS), where the analytical current distribution is needed [1,2,3,4,5,6,7]. Two promising approximate analytical methods for FSS are the periodic method of moment (PMM) and multimodal network approach. However, it is necessary for both of them to obtain the current/field distribution in the scatterer. PMM solves this problem using MoM [8,9,10], while the multimodal network approach only considers simple rectangular scatters [11,12,13]. For a rectangular patch (or aperture) under oblique incidence in the principle scan plane of the structure, approximate closed-form expressions for aperture field and current profiles of patch surface were obtained. However, to the authors’ knowledge, the approximate closed-form expressions for other structures have not been reported. Therefore, the application of the above approximate analytical methods depends on the development of current/field distribution in the scatterer of other types. In [14], the spatial current/field profile is calculated using a full-wave simulation, at a single- and low-frequency value. It is then assumed to be independent of the frequency in the considered range of interest. However, this method is restrictive, treating structures that are relatively simple and incidence angles that are relatively small. Besides, the numerical preparation is time consuming and means the analytical method is complex.

The calculation of spatial current/field profile for an FSS element is similar to the analysis of current distribution for line antenna [15,16]. The equivalent transmission line model was often adopted for the study of input impedance and mutual coupling of transmitting line antenna [17,18,19]. It was usually a two-wire transmission line model with lossy lumped feed. For single antennas, radiation lossy can be considered easily, as can the mutual coupling lossy. A two-wire straight antenna refers to two collinear or equidistant antennas, which can be one or more segments. The segments may or may not be parallel. The two-wire straight antenna can only be equivalent to the two-wire transmission line model, and the requirement is that the antenna structures are symmetrical. Besides, the tangential component of the incident electric field on the two antennas should be even or odd symmetrical. For the asymmetric distribution of the incident field, the incident field can be decomposed into odd and even symmetrical. The total current in the antenna is then the superposition of currents in the above two modes.

Schelkunoff mainly used a one-wire transmission line model in [17] to study the input impedance, mutual coupling, antenna impedance, and the approximate solution of transmitting/receiving antenna current. Compared to the two-wire transmission line model, the one-wire transmission model is more flexible. The former makes it easier to study the dipole oscillator antenna. For the one-wire transmission line model of complex structures, the main problem is that the equations and parameters are restrictive in approximating the lossless case. Schelkunoff also studied the current distribution on the receiving antenna (actually a reflector antenna, which is a straight continuous fine wire antenna, which is exposed to the incident field and the corresponding tangential component is uniform) using a two-wire transmission line model. The model is restrictive for vertical incidence (the tangential field of incident field is uniform on the antenna). For oblique incidence, the tangential electric field of the incident field on the antenna varies with the spatial position. It results in the induced electromotive force distributed asymmetrically about the center of the antenna. The currents on the lines are not equivalent and reverse, so the two-wire model cannot be used.

In this paper, the current distributions of transmitting and receiving antenna, based on the equivalent transmission line method, are analyzed. Both the two-wire and one-wire model are studied, together with the radiation lossy. An iterative scheme is then introduced to further improve the accuracy. The current distribution calculated is in analytic form, which can be directly used to approximate the analytical method for FSS.

## 2. Transmitting Antenna

### Equivalent Circuit of the Transformers

**Figure 1:**
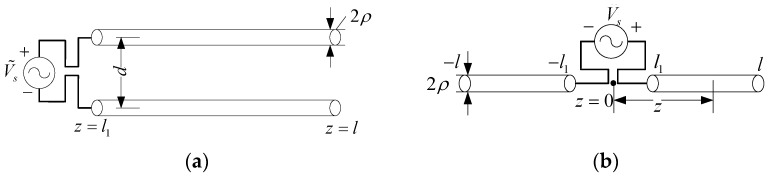
Symmetric diploes transmitting line antenna (**a**) equidistance; (**b**) collinear.

**Figure 2:**
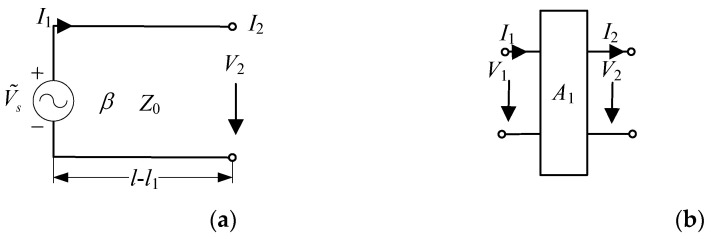
Two-wire equivalent transmission line model of Figure 1. (**a**) Equivalent transmission line; (**b**) cascaded two-port network.

Symmetric diploes transmitting antenna (Figure 1) can be equivalent to the two-wire transmission line model (Figure 2a). For the lossless equivalent transmission line corresponding to equidistance symmetric diploes antenna (Figure 1a), the average characteristic impedance is evaluated using [14,15]

(1) $$Z_{0}=\frac{120}{\sqrt{\varepsilon_{r}}}\ln\frac{d}{\rho }.$$

For the lossless equivalent transmission line corresponding to collinear symmetric diploes antenna (Figure 1b), the average characteristic impedance is calculated as [17,18]

(2) $$Z_{0}=\frac{1}{l-l_{1}}\int_{l_{1}}^{l}\frac{120}{\sqrt{\varepsilon_{r}}}\ln\frac{2\left(z-l_{1}\right)}{\rho }dz.$$

The per-unit-length inductance and capacity can then be obtained

(3) $$L=\sqrt{\mu_{0}\varepsilon_{0}\varepsilon_{r}}Z_{0},\;C=\frac{\sqrt{\mu_{0}\varepsilon_{0}\varepsilon_{r}}}{Z_{0}}.$$

The propagation constant is $r_{1}=j\beta =j\omega \sqrt{LC}=j\omega \sqrt{\mu_{0}\varepsilon_{0}\varepsilon_{r}}$.

According to the theory of the transmission line [20,21], the equivalent transmission line model in Figure 2a can be equivalent to the cascaded two-port network in Figure 2b. *V*_i_ and *I*_i_ denote the corresponding port voltage and current, respectively. A voltage source $V_{1}={\tilde{V}}_{s}$ is connected at the left-hand-side port and the right-hand-side is an open circuit ($I_{2}=0$). The transmission matrix *A*_1_ can be expressed as

(4) $$A_{1}=\left[\begin{array}{cc} \cos\left[\beta \left(l-l_{1}\right)\right] & -jZ_{0}\sin\left[\beta \left(l-l_{1}\right)\right]\\ \frac{\sin\left[\beta \left(l-l_{1}\right)\right]}{jZ_{0}} & \cos\left[\beta \left(l-l_{1}\right)\right] \end{array}\right].$$

According to the microwave network theory, we obtain

(5) $$\left[\begin{array}{c} V_{2}\\ I_{2} \end{array}\right]=A_{1}\left[\begin{array}{c} V_{1}\\ I_{1} \end{array}\right].$$

Substituting the boundary condition $V_{1}={\tilde{V}}_{s}$ and $I_{2}=0$ into Equation (5), we can obtain the coefficients of the voltages and currents

(6) $$V_{2}=\frac{{\tilde{V}}_{s}}{\cos\left[\beta \left(l-l_{1}\right)\right]},\;I_{1}=j\tan\left[\beta \left(l-l_{1}\right)\right]\frac{{\tilde{V}}_{s}}{Z_{0}}.$$

Based on the above port voltages and currents, the currents distribution of the equivalent transmission lines (Figure 1) can be written as

(7) $$I\left(z\right)=I_{1}\cos\left[\beta \left(z-l_{1}\right)\right]+\frac{V_{1}}{jZ_{0}}\sin\left[\beta \left(z-l_{1}\right)\right].$$

**Figure 3:**
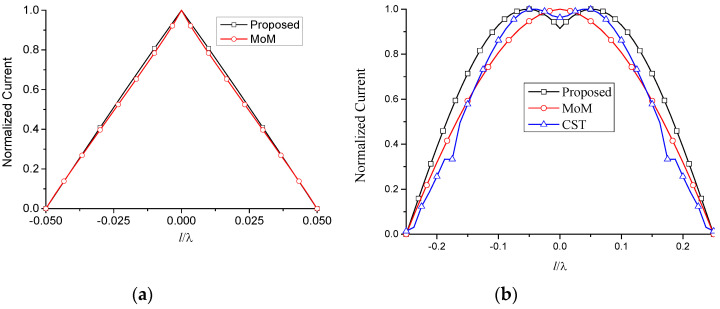
Normalized current distribution of the proposed method and MoM. (**a**) l=0.05λ; (**b**) l=0.25λ.

The simulated normalized current distribution of the proposed method and MoM are shown in Figure 3. It can be observed that the results comply well with each other when l=0.05λ. However, they differ from each other when l=0.25λ. In fact, there is deviation for MoM when $\varepsilon_{r}\neq 1.0$. It should be pointed out that this method can be extended to multi-section line antenna. In this case, the equivalent transmission line model and two-port network are cascaded by the corresponding multi sections. It would affiliate the analysis of the complicated line antenna.

## 3. Two-Wire Transmission Line Model of Receiving Antenna

**Figure 4:**
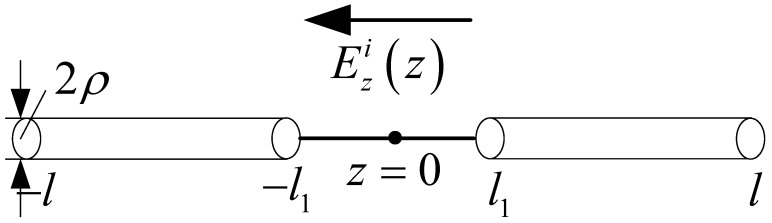
Two-wire straight antenna exposed to incident electric field.

As discussed in the introduction, the necessary condition for a single straight antenna to be equivalent to a two-wire transmission line model is that the incident field of the antenna is symmetrical. Mode decomposition can be applied to asymmetric situations. In this section, the equivalent transmission model is applied to the analysis of receiving antenna, shown in Figure 4.

**Figure 5:**
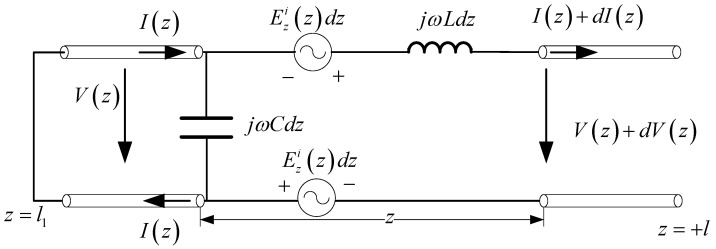
Two-wire transmission line model of Figure 4.

The two-wire lossless transmission line model of Figure 4 is shown in Figure 5, where V(z) is the voltage between the two lines. The voltage and current on the line are governed by the transmission line equations

(8) $$\frac{\partial V\left(z\right)}{\partial z}=-jwL\cdot I\left(z\right)+2E_{z}^{i}\left(z\right)$$

(9) $$\frac{\partial I\left(z\right)}{\partial z}=-jwC\cdot V\left(z\right)$$

The corresponding transmission line parameters can be calculated following Section 2. The boundary condition is

(10) I(−l)=I(+l)=0.

Substituting (10) into (8) and (9) yields the general expression of potential and current

(11) $$\begin{array}{l} V\left(z\right)=Ae^{-r_{1}z}+Be^{r_{1}z}+\varphi_{v}\left(z\right)\\ I\left(z\right)=\frac{1}{Z_{0}}\left[Ae^{-r_{1}z}-Be^{r_{1}z}+\varphi_{i}\left(z\right)\right] \end{array}$$

$\varphi_{v}\left(z\right)$ and $\varphi_{i}\left(z\right)$ are the terms corresponding to $E_{z}^{i}\left(z\right)$. The coefficients A and B can be obtained according to the boundary condition of the transmission line. Suppose the incident field on the antenna is

(12) $$E_{}^{i}\left(z\right)=E_{0}\cos\theta \cdot e^{-j\beta_{0}\sin\theta z}.$$

where $\beta_{0}=j\omega \sqrt{\mu_{0}\varepsilon_{0}}$, θ is the incident angle. Since the incident field is asymmetrically distributed, mode decomposition is required.

(13) $$E_{e}^{i}\left(z\right)=\frac{E_{}^{i}\left(z\right)+E_{}^{i}\left(-z\right)}{2}=E_{0}\cos\theta \cdot \cos\left(\beta_{0}\sin\theta z\right)$$

(14) $$E_{o}^{i}\left(z\right)=\frac{E_{}^{i}\left(z\right)-E_{}^{i}\left(-z\right)}{2}=-jE_{0}\cos\theta \cdot \sin\left(\beta_{0}\sin\theta \cdot z\right)$$

**Figure 6:**
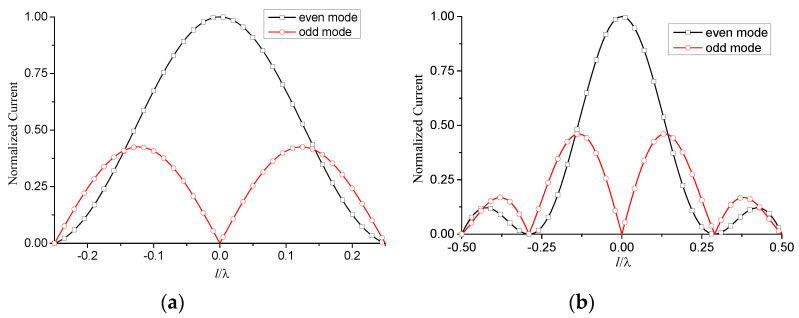
Normalized current distribution of two-wire transmission line model. $l_{1}=0,\;\rho =1\times 10^{-7}\;m,\;f=10\;\mathrm{GHz},\;\varepsilon_{r}=1.6,\;\theta =0^{{}^{\circ}}$. (**a**) l=0.25λ; (**b**) l=0.5λ.

The total current on the antenna is then obtained by the superposition of the above two modes. The corresponding simulated results are shown in Figure 6.

## 4. One-Wire Transmission Line Model of Receiving Antenna

**Figure 7:**
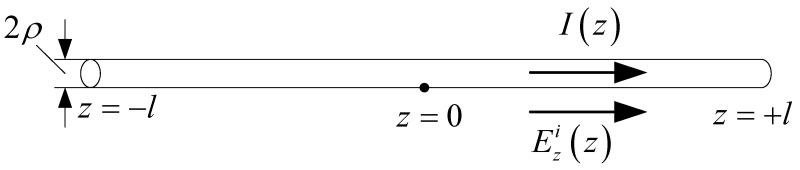
Single straight antenna exposed to incident electric field.

**Figure 8:**
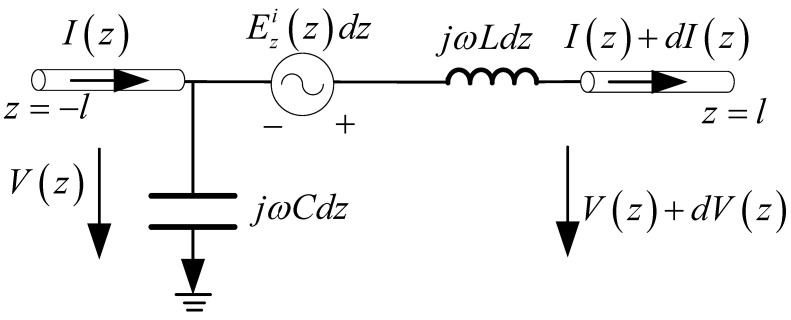
One-wire equivalent transmission line model of Figure 7.

In this section, the one-wire transmission line model for a single straight antenna is analyzed. In this case, the mode decomposition of the incident field is eliminated. A single straight antenna is a continuous straight-line antenna, which could be one section or multiple non-parallel sections. It can be equivalent to the one-wire equivalent transmission line model. Each section corresponds to a potential and a current equation. A single straight antenna with one section is illustrated in Figure 7, and $E_{z}^{i}\left(z\right)$ is the tangential component in the incident electric field. The corresponding one-wire equivalent transmission line model is shown in Figure 8. For the transmission line excited by the distribution voltage source, the electric potential and current on the line are governed by the transmission line equations.

(15) $$\frac{\partial V\left(z\right)}{\partial z}=-jwL\cdot I\left(z\right)+E_{z}^{i}\left(z\right)$$

(16) $$\frac{\partial I\left(z\right)}{\partial z}=-jwC\cdot V\left(z\right)\;$$

V(z) and I(z) are potential and current, respectively. The boundary condition is

(17) I(−l)=I(+l)=0

**Figure 9:**
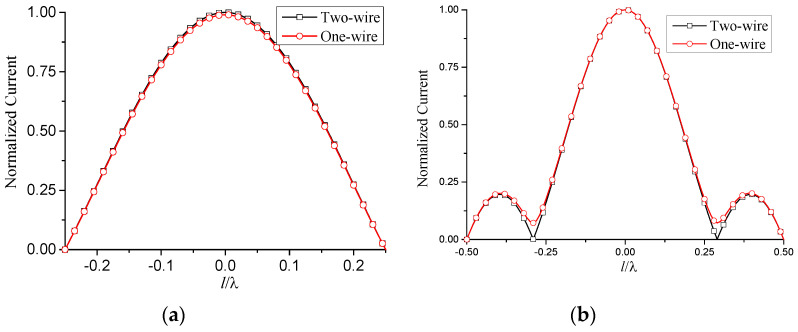
Normalized current distribution of one-wire transmission line model. $l_{1}=0,\;\rho =1\times 10^{-7}\;m,\;f=10\;\mathrm{GHz},\;\varepsilon_{r}=1.6,\;\theta =0^{{}^{\circ}}$. (**a**) l=0.25λ; (**b**) l=0.5λ.

Compared with the two-wire equivalent transmission line model, the one-wire model is a more general method. It can be used for any antenna composed of relatively narrow strips or slots. This will benefit the analysis of FSS, where the element currents are usually unknown. The normalized current distribution of Figure 7 is shown in Figure 9, together with the results of the two-wire model.

## 5. Loss of Equivalent Transmission Line

The equivalent transmission line models discussed above are lossless. In this section, the antenna lossy is analyzed. In this case, the current in the receiving antenna needs to be obtained first and the per unit length radiation resistance can then be calculated. However, the current is practically an unknown quantity. One way to obtain it is to approximate the current in the form of sine function. However, it is restricted to some special cases (e.g., electrically small antenna and vertical incident). An alternative way is to use the lossless receiving current $I_{0}\left(z\right)$ as the approximation, which has been discussed in Section 3 and Section 5. $I_{0}\left(z\right)$ produces the scattered fields, namely the re-radiated field. The radiation energy is the radiation lossy of the antenna. Consequently, the radiation resistance, potential and current of the equivalent transmission line model can then be solved. Through the iterative algorithm of the above process, a more accurate current distribution of the receiving antenna can be obtained.

Suppose r is parallel to $r_{1}$, $\theta =\theta_{1}$. When calculating the magnitude of the field, assume $r_{1}\approx r$. When calculating the phase of a field, assume $r_{1}\approx r-z\cos\theta$. The far-field radiation produced by the current element is

(18) $$dE_{\theta }\approx j\frac{30\beta }{\sqrt{\varepsilon_{r}}}\frac{I\left(z\right)dz}{r}\sin\theta e^{-j\beta \left(r-z\cos\theta \right)}$$

The far-field radiation electric field of the antenna is the integral of (18) on the whole antenna

(19) $$E_{\theta }=\int_{-l}^{l}dE_{\theta }dz=j\frac{30\beta }{\sqrt{\varepsilon_{r}}}\frac{e^{-j\beta r}}{r}\sin\theta \int_{-l}^{l}I\left(z\right)e^{j\beta z\cos\theta }dz.$$

According to the approximation property of the plane wave in the far field, the active power of the antenna is

(20) $$P_{r}=\frac{1}{2}\oint_{S}\left[\vec{E}\times {\vec{H}}^{*}\right]\cdot d\vec{s}=\frac{1}{2}\int_{0}^{2\pi }\int_{0}^{\pi }\frac{1}{Z_{w}}{\left|E_{\theta }\right|}^{2}r^{2}\sin\theta d\theta d\varphi$$

where $Z_{w}=120\pi /\sqrt{\varepsilon_{r}}$. Substituting (19) into (20) yields

(21) $$P_{r}=\frac{5\pi \beta^{2}}{\sqrt{\varepsilon_{r}}}\int_{0}^{\pi }{\left|\int_{-l}^{l}I\left(z\right)e^{j\beta z\cos\theta }dz\right|}^{2}\sin^{3}\theta d\theta$$

**Figure 10:**
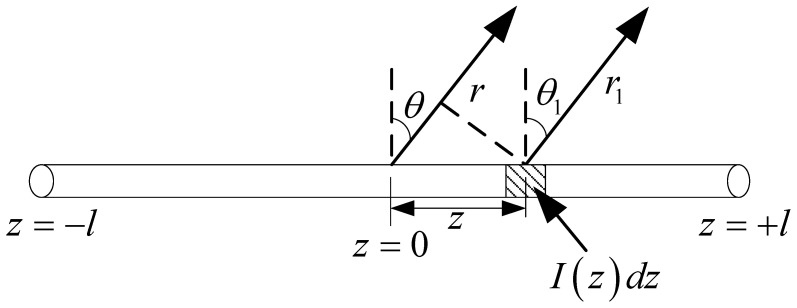
The radiation field of the receiving antenna current.

The total lossy on the transmission line (Figure 10, the average actual power) is

(22) $$P_{r}{}^{\prime }=\frac{1}{2}\int_{-l}^{l}{\left|I(z)\right|}^{2}R_{1}dz$$

Suppose that all radiation lossy of an antenna are equivalent to the total power dissipated on the per unit length radiation resistance $R_{1}$ of the transmission line, we can solve $R_{1}$

(23) $$R_{1}=\frac{\frac{15\beta_{r}^{2}}{\sqrt{\varepsilon_{r}}}\int_{0}^{\pi }{\left|\int_{-l}^{l}I\left(z\right)e^{j\beta z\cos\theta }dz\right|}^{2}\sin^{3}\theta d\theta }{\int_{-l}^{l}\left|I\left(z\right)\right|dz}$$

**Figure 11:**
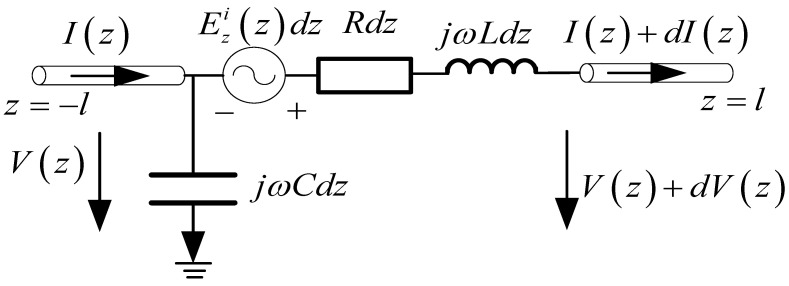
One-wire equivalent lossy transmission line model of Figure 7.

Consequently, the equivalent transmission line model of Figure 2, Figure 5 and Figure 8 should be revised. Taking Figure 7 as an example, the lossy is shown in Figure 11. The corresponding transmission line equations and parameters should be revised as well

(24) $$\frac{\partial V\left(z\right)}{\partial z}=-\left(jwL+R_{1}\right)\cdot I\left(z\right)+E_{z}^{i}\left(z\right)$$

(25) $$\frac{\partial I\left(z\right)}{\partial z}=jwC\cdot V\left(z\right)$$

(26) $$Z_{c}=\sqrt{\left(jwL+R_{1}\right)/jwC}$$

(27) $$r=j\beta_{r}=\sqrt{\left(jwL+R_{1}\right)j\omega C}=j\omega \sqrt{\mu_{0}\varepsilon_{0}\varepsilon_{r}}$$

**Figure 12:**
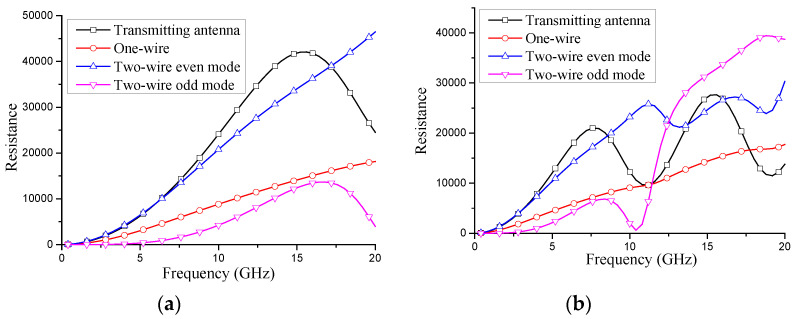
Per unit length radiation resistance. (**a**) l=0.25λ; (**b**) l=0.5λ.

The simulation results show that the lossy has little influence on the normalized current distribution. The per unit length radiation resistance $R_{1}$ is shown in Figure 12.

## 6. Conclusions

The equivalent transmission line model is established for the current analysis of line antenna. The two-wire model is discussed for transmitting antenna and receiving antenna (the incident field should be decomposed for asymmetric case). The one-wire model is introduced for any receiving antenna composed of narrow strips. The corresponding mode decomposition is eliminated. The transmission line radiation resistance hardly effects the normalized current distribution.
